# Supplementary material for: Features of repertoire diversity and gene expression in human cytotoxic T cells following allogeneic hematopoietic cell transplantation
Source: Commun Biol. 2021 Oct 11;4:1177. doi: 10.1038/s42003-021-02709-7 (PMC8505416; doi:10.1038/s42003-021-02709-7)
Supplement: Supplementary file 2 — Supplementary information [file 42003_2021_2709_MOESM2_ESM.pdf]

Supplementary information for

Features of repertoire diversity and gene expression in human cytotoxic T cells following allogeneic hematopoietic cell transplantation

Authors:
Hideki Nakasone[1*], Machiko Kusuda[1], Kiriko Terasako-Saito[1], Koji Kawamura[1], Yu Akahoshi[1], Masakatsu Kawamura[1], Junko Takeshita[1], Shunto Kawamura[1], Nozomu Yoshino[1], Kazuki Yoshimura[1], Yukiko Misaki[1], Ayumi Gomyo[1], Kazuaki Kameda[1], Aki Tanihara[1], Masaharu Tamaki[1], Shun-ichi Kimura[1], Shinichi Kako[1], and Yoshinobu Kanda[1*]
Affiliations:

1 Division of Hematology, Jichi Medical University Saitama Medical Center, Saitama, Japan

*Correspondence:
Yoshinobu Kanda, MD/PhD / ycanda-tky@umin.ac.jp and
Hideki Nakasone, MD/PhD / nakasone-tky@umin.ac.jp

Division of Hematology, Jichi Medical University Saitama Medical Center,
1-847 Amanuma-cho Omiya-ku, Saitama, 330-8503, Japan
Tel: +81-48-647-2111

Supplementary Table 1. Smarter-NGS method for TCR determination.

1. Sorting preparation	Components	µL / a PCR tube	
	D-PBS (-) (FUJI FILM-Wako)	2.5	*SMARTer® Human TCR a/b Profiling Kit (Takara bio)
	10xLysis buffer (19µL) * & RNAse inhibitor (1µL) *	1.0	
	or<br>RNAse-free H2O with 0.5% Triton X (nacalai tesque)(19µL) and RNAse inhibitor (1µL) *	3.5	
	total volume	3.5	
2. Sorting	After sorting cells into a PCR tube per a sample, add RNAse-free H2O, and make a volume of 10.5ul in total.		
3. RT reaction mix preparation	Components	µL / a PCR tube	
	Sorted sample (above)	10.5	*SMARTer® Human TCR a/b Profiling Kit (Takara bio)
	TCR dT primer*	2.0	
	total volume	12.5	
	72°C 3 min → 4°C 2 min		
	Components		
	Heated sample (above)	12.5	
	5x Ultra Low First-Strand buffer *	4.0	
	SMART-seq v4 Oligonucleotide *	1.0	
	RNAse inhibitor*	0.5	
	SMARTScribe Reverse Transcriptase*	2.0	
	total volume	20.0	
4. RT reaction	42°C 45 min → 70°C 10 min → 4°C ∞		
5. PCR1 reaction mix preparation	Components	µL / a PCR tube	
	2x SeqAmp PCR CB buffer (Takara bio)	25.0	*SMARTer® Human TCR a/b Profiling Kit (Takara bio)
	SMART primer 1 *	0.5	
	TCRb Human primer *	0.5	
	SeqAmp DNA Polymerase *	1.0	
	RNAse-free H2O	3.0	
	RT template	20.0	
	Total volume	50	
6. PCR1	95°C 1 min → [94°C 1 min → 53°C 1 min→ 68°C 1 min] x 30 cycles → 72°C 10 min → 4°C ∞		

7.	PCR2 reaction mix preparation	Components	μL for a PCR tube	
		2x SeqAmp PCR CB buffer (Takara bio)	25.0	*SMARTer® Human TCR a/b Profiling Kit (Takara bio)
		TCRb reverse primer 2 *	0.5	
		SeqAmp DNA Polymerase *	1.0	
		RNAse-free water	22.0	
		TCRb forward primer 2 *	0.5	
		PCR1 product	1	
		Total volume	50	
8.	PCR2	95°C 1 min → [94°C 1 min → 53°C 1 min→ 68°C 1 min] x 20 cycles → 72°C 10 min → 4°C ∞		
9.	SPRI purification & Quality check	Purification with AmpureXp Beads (Beckman Coulter) according to the manual for the SMARTer® Human TCR a/b Profiling Kit (Takara bio).<br>Quality check using Agilent Bioanalyzer 2100 (Agilent Technologies) with Agilent DNA 1000 reagent (Agilent Technologies).		

Supplementary Table 2. Direct single-cell method for TCR determination

Step	Components	μL / a PCR tube	Notes
1. RT reaction Mix preparation	5X First strand buffer* (with 0.5% Triton X)	3.00	* SuperScript III reverse transcriptase kit (invitrogen)
	10μM 3' BC-RT primer #	0.30	
	25mM dNTP (Invitrogen)	0.30	
	Rnase Inhibitor (nacalai tesque)	0.38	
	SuperScript III reverse transcriptase *	0.34	# Primer list (Cancer Sci 2010; 101: 594–600)
	1mg/ml gelatin (Roche)	1.50	
	1mg/ml tRNA (MERCK)	1.50	
	RNAse-free H2O	7.68	
	total volume	15.0	
2. Sorting & RT reaction	50°C 90 min → 95°C 5 min → 4°C ∞		
3. PCR1 reaction mix preparation	Components	μL / a PCR tube	
	10X PCR buffer (without Mg2+) *	5.00	* Platinum Taq DNA polymerase kit (invitorgen)
	25mM MgCl2 (ROSHE)	4.00	
	10mM dNTP (Invitrogen)	1.25	
	Platinum Taq DNA polymerase *	0.20	
	RNAse-free H2O	26.05	# Primer list (Cancer Sci 2010; 101: 594–600)
	0.5uM 3' BC-RT primer #	0.50	
	BV primer mix1 (0.25 μM in each: TRBV9/5, 25 10, 20, 28, 2, 6, 24) #	1.00	
	BV primer mix2 (0.25 μM in each: TRBV29, 7, 27, 7-8, 12, 11, 13, 15) #	1.00	
	BV primer mix3 (0.25 μM in each: TRBV 19,30,4,3,18, 21, 14, 23) #	1.00	
	total mixture	40.0	
	RT template volume	10.0	
	Total reaction volume	50.0	
4. PCR1	95 °C 2 min → [94 °C 45 sec → 57°C 45 sec → 72°C 1 min] x 40 cycles → 72°C 7 min → 4°C ∞		
5. PCR2 reaction mix preparation	Components	μL for 100 wells	
	10X PCR buffer (without Mg2+) *	250	* Platinum Taq DNA polymerase kit (Invitorgen)
	50 mM $MgCl_2$ *	125	
	10mM dNTP (Invitrogen)	50	

	Platinum Taq DNA polymerase *	6.25	
	RNAse-free H2O	1931	# Primer list (Cancer Sci 2010; 101: 594–600)
	10μM 5' BC primer #	37.5	
	total mixture volume	2400.0	
		↓	
	Dispense 294 μL of the mixture above into 8 tubes (S1-S8)		
	Add primers below	↓	
	S1 (TRBV9/5, 25 10: 15μM in each) #	6	→ (S1) 300 μL in total
	S2 (TRBV20, 28, 2: 15μM in each) #	6	→ (S2) 300 μL in total
	S3 (TRBV29, 7, 27: 15μM in each) #	6	→ (S3) 300 μL in total
	S4 (TRBV7-8, 12, 11: 15μM in each) #	6	→ (S4) 300 μL in total
	S5 (TRBV 19,30,4,3,18: 15μM in each) #	6	→ (S5) 300 μL in total
	S6 (BV21, 14, 23: 15μM in each) #	6	→ (S6) 300 μL in total
	S7 (TRBV6, 24: 15μM in each) #	6	→ (S7) 300 μL in total
	S8 (TRBV13, 15: 15μM in each) #	6	→ (S8) 300 μL in total
		↓	
	Dispense 24 μL of the individual mixture (S1-8) in 12 wells in each on a 96-well plate and suspend 1μL of PCR1 product individually in 8 wells of S1- S8 on a 96-well plate		
	total mixture in each well	24.00	
	PCR1 product in each well	1.00	
	reaction volume in each well	25.00	
6. PCR2	95 °C 2min → [94 °C 45 sec → 57°C 45 sec → 72°C 50 sec] x 35-37 cycles → 4°C ∞		
7. Electrophoresis	Determine the BV family usage (S1, S2, … or S8)		
8. PCR3 reaction mix preparation	Components	μL for 100 wells	
	10X PCR buffer (without Mg2+) *	250	* Platinum Taq DNA polymerase kit (invitorgen)
	50 mM MgCl2 *	125	
	10mM dNTP (Invitrogen)	50	
	Platinum Taq DNA polymerase*	6.25	
	RNAse-free H2O	1931	# Primer list (Cancer Sci 2010; 101: 594–600)
	10μM 5' BC primer #	37.5	
	total mixture in each well	24.00	
	PCR1 product in each well	1.50	
	Identified specific-BV primer in each well #	1.00	
	reaction volume in each well	26.50	

9. PCR3	95 °C 2min → [94 °C 45 sec → 57°C 45 sec → 72°C 50 sec] x 35-37 cycles → 4°C ∞
10. Electrophoresis & purification	Determine the specific BV usage
11. Preparation for sequencing	Components / μL for 1 well<br>5X sequencing buffer * — 3.00<br>BigDye® Terminator v3.1 * — 0.90<br>RNAse-free H2O — 14.10<br>total mixture (above) — 18.00<br>PCR3 product — 1.00<br>Vb-specific primer — 1.00<br>Total volume — 20.00<br>* BigDye® Terminator v3.1 Cycle Sequencing kit (ABI)
12. Thermal cycle	96 °C 1min → [96°C 10 sec → 50°C 5 sec → 60°C 40 sec] x 25-26 cycles → 4°C ∞
13. Purification & sequence	Sequence using Applied Biosystems 3130/3130Xl Genetic Analyzer (Applied Biosystems)

Nested table for step 11:

Components	μL for 1 well	
5X sequencing buffer *	3.00	* BigDye® Terminator v3.1 Cycle Sequencing kit (ABI)
BigDye® Terminator v3.1 *	0.90	
RNAse-free H_2O	14.10	
total mixture (above)	18.00	
PCR3 product	1.00	
Vb-specific primer	1.00	
Total volume	20.00	

Supplementary Table 3. Smarter RNA-sequencing method

Step	Components	µL / a PCR tube	
1. Sorting preparation	Components	µL / a PCR tube	
	RNAse-free H2O with 0.5% Triton X (nacalai tesque)(19µL) and RNAse inhibitor (1µL) *	3.5	*SMART-Seq® v4 Ultra® Low Input RNA Kit (Takara bio)
	total volume	3.5	
2. Sorting	After Sorting cells into a PCR tube per a sample, add RNAse-free H2O make a volume of 10.5ul in total.		
3. RT reaction mix preparation	Components	µL / a PCR tube	
	Sorted sample (above)	10.5	*SMART-Seq® v4 Ultra® Low Input RNA Kit (Takara bio)
	3' SMART-Seq CDS Primer II A† *	2.0	
	total volume	12.5	
	72°C 3 min → 4°C 2 min		
	Components		
	Heated sample (above)	12.5	
	5X Ultra Low First-Strand Buffer *	4.0	
	SMART-Seq v4 Oligonucleotide *	1.0	
	RNAse inhibitor *	0.5	
	SMARTScribe Reverse Transcriptase *	2.0	
	total volume	20.0	
4. RT reaction	42°C 90 min → 70°C 10 min → 4°C ∞		
5. PCR reaction mix preparation	Components	µL / a PCR tube	
	2x SeqAmp PCR CB buffer (Takara bio)	25.0	*SMART-Seq® v4 Ultra® Low Input RNA Kit (Takara bio)
	PCR Primer II A *	1.0	
	SeqAmp DNA Polymerase *	1.0	
	RNAse-free H2O	3.0	
	RT template	20.0	
	Total volume	50	
6. cDNA Amplification by LD-PCR	95°C 1 min → [98°C 10 sec → 65°C 30 sec→ 68°C 3 min] x16 cycles → 72°C 10 min → 4°C ∞		
7. SPRI purification & Quality check	Purification with AmpureXp Beads (Beckman Coulter) according to the manual for the SMART-Seq® v4 Ultra® Low Input RNA Kit (Takara bio).<br>Quality check using Agilent Bioanalyzer 2100 (Agilent Technologies) with Agilent High Sensitivity DNA kit (Agilent Technologies).		

Supplementary Figure 1.

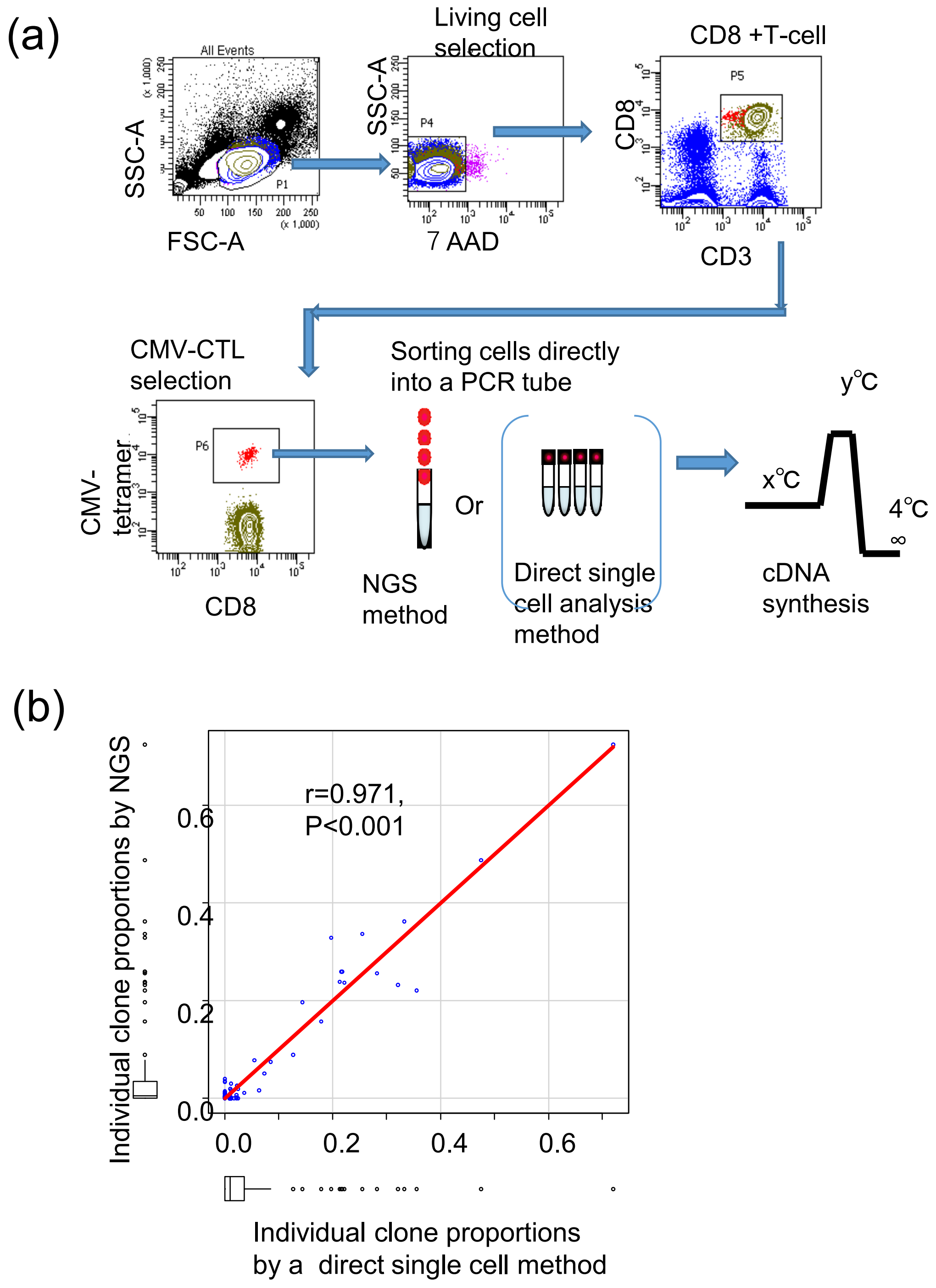

Supplementary Figure 1. A simple scheme for identification of HLA-A24-restricted CMV-pp65-specific cytotoxic T-cells (CMV-CTLs) and correlation of the current next-generation sequence (NGS) strategy and our previous direct single-cell method.

(a) Identification of CMV-CTLs and difference in sorting between the methods. CMV-CLTs were defined as CD3+CD8+HLA-A*24-CMV-pp65 (QYDPVAALF)-tetramer+ T-cells. (b) Correlation between individual clone proportions identified by the current NGS strategy and our previous direct single-cell method (r=0.971, P<0.001 by Pearson's correlation test, n=5 samples).

Supplementary Figure 2.

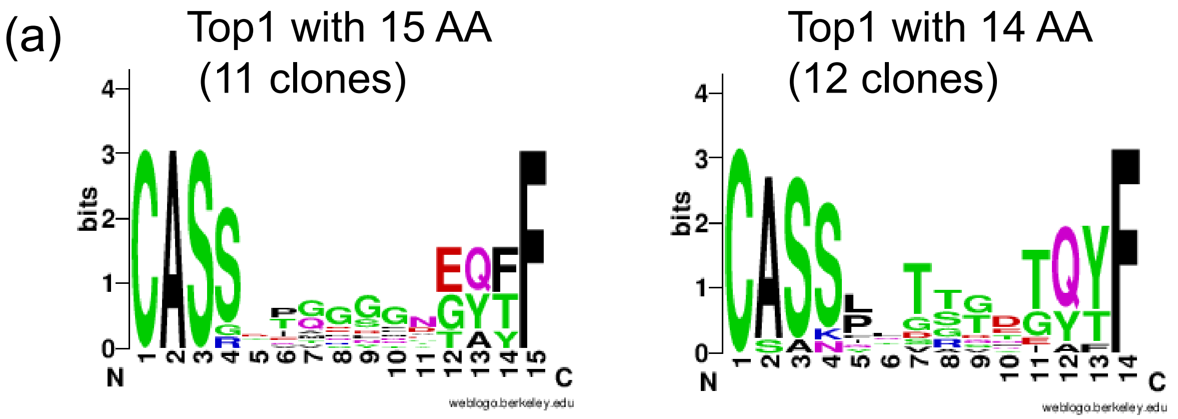

** Top1 clones with <14 or >15 were too small to create sequence logos.

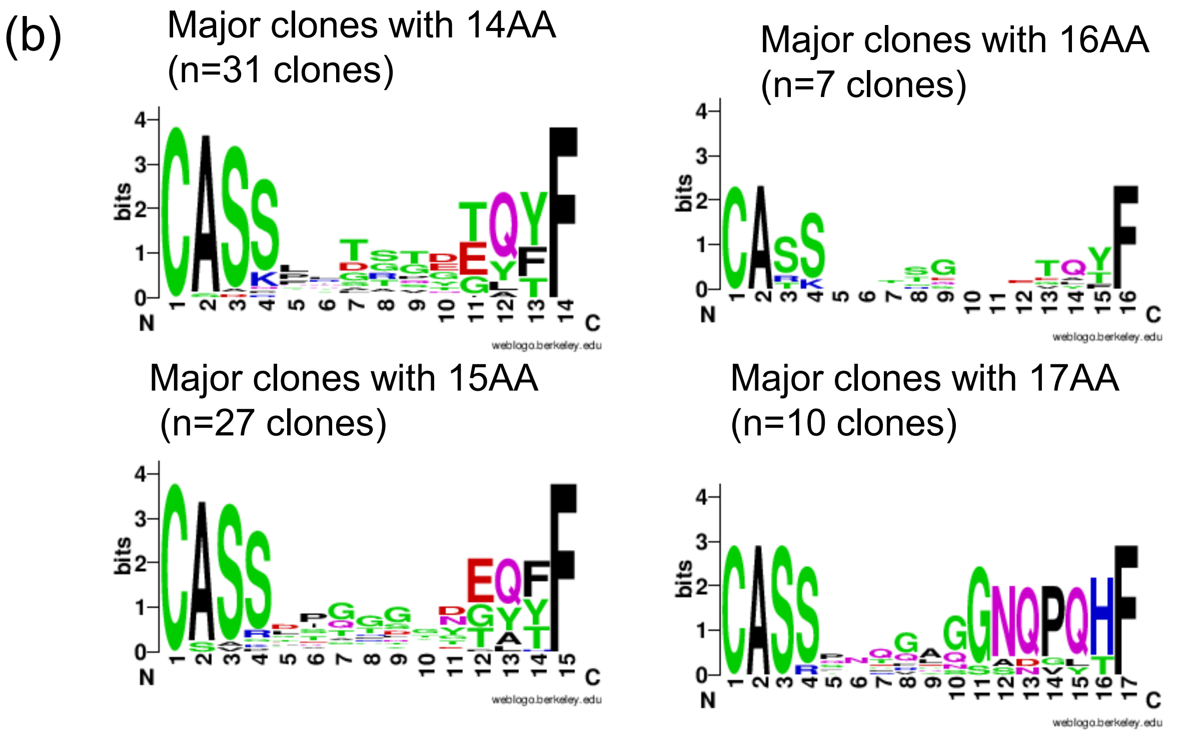

Supplementary Figure 2. Amino acid (AA) sequence logos of the top 1 or major clones.

(a) AA sequence logos of the top 1 clones of HLA-A24-restricted CMV-pp65-specific cytotoxic T-cells (CMV-CTL). Logos of 15 and 14 AA of TCRβ-CDR3 are shown, but there were too few top 1 clones with >15 or <14 AA to create sequence logos. (b) AA sequence logos of TCRβ-CDR3 according to AA length in major CMV-CTLs accounting for >5% of all CMV-CTLs within individual recipients in the early or late phases after allo-HCT. X-axis denotes the position of amino acids from the N- to C-terminal.

Supplementary Figure 3

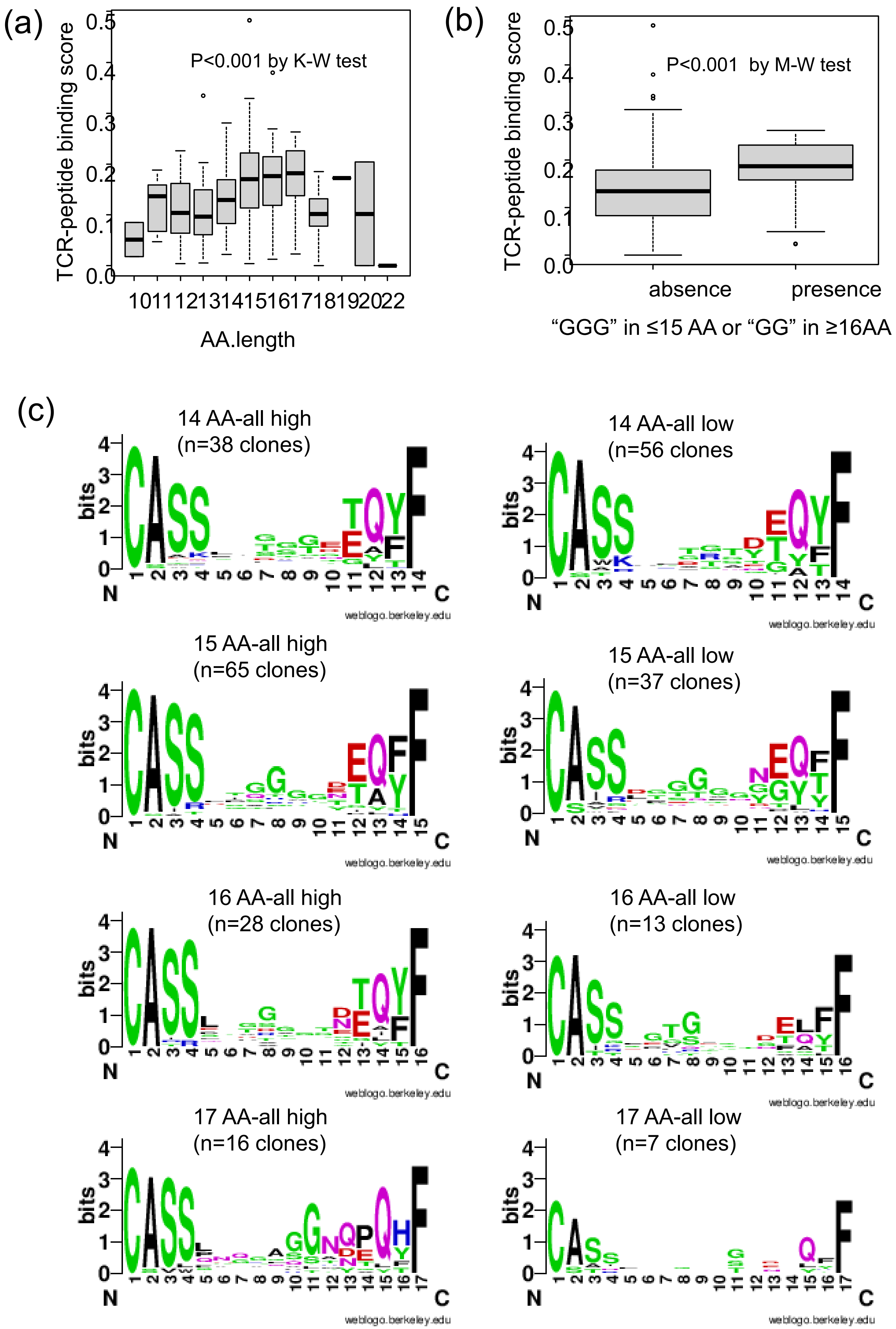

Supplementary Figure 3. TCR-peptide binding scores in all of HLA-A24-restricted CMV-pp65-specific cytotoxic T-cell (CMV-CTL) clones.

(a) TCR-peptide binding scores according to amino acid (AA) length ($P<0.001$ by the Kruskal-Wallis (K-W) test). (b) TCR-peptide binding scores between the CMV-CTL clones with and without the “GGG” motif in ≤15 AA or “GG” motif in ≥16 AA in their TCRβ-CDR3 ($P<0.001$ by the Mann-Whitney U (M-W) test). (c) AA sequence logos in CMV-CTLs with higher and lower binding scores to the CMVpp65 peptide according to AA length of CDR3-TCRβ. A score higher than a median value of 0.141 was defined as a higher binding score. Individual box and whisker plots were constructed by the 25th percentile (Q1), median, and 75th percentile (Q3) with whiskers of 1.5 times interquartile range (IQR) lengths.

Supplementary Figure 4

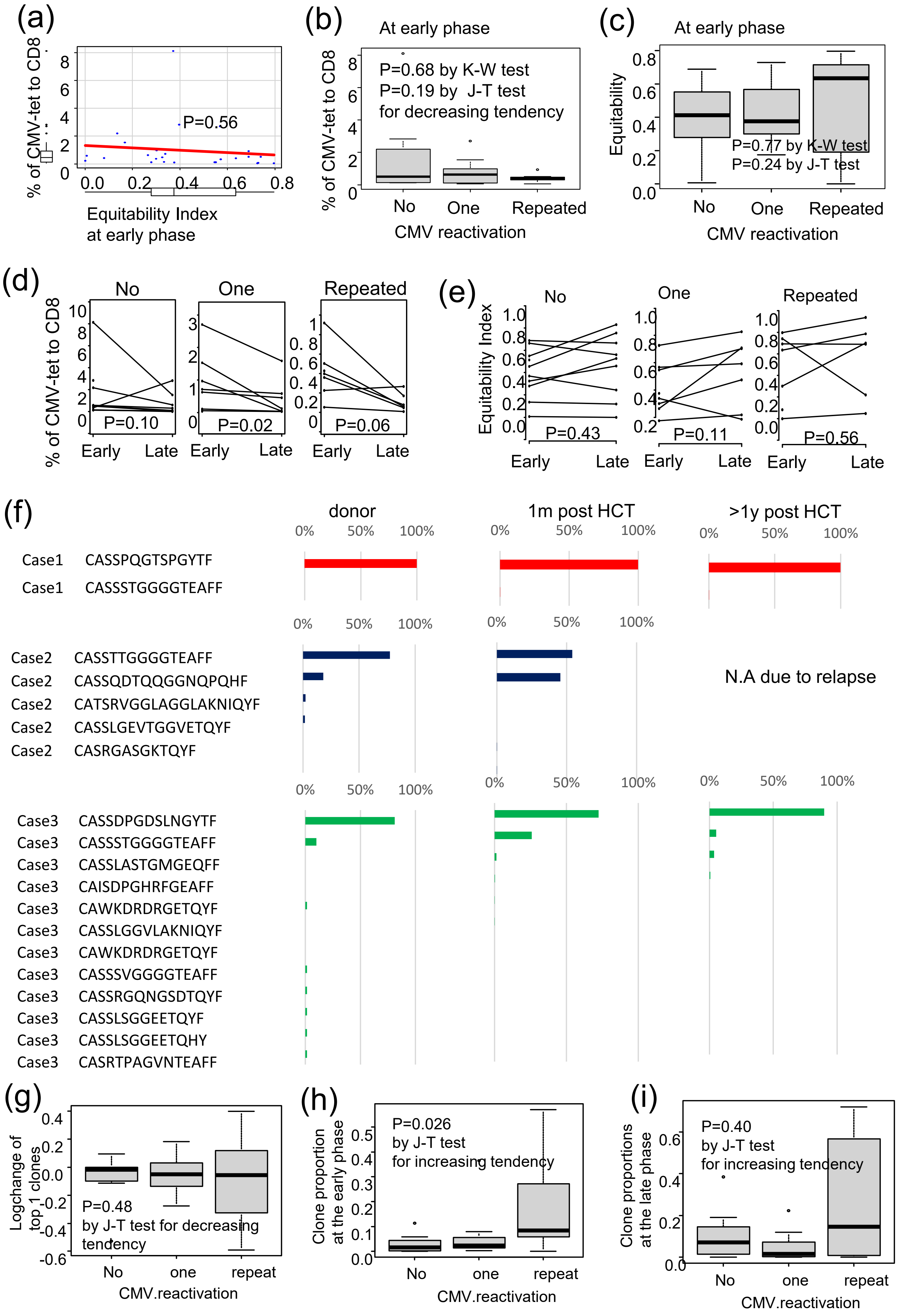

Supplementary Figure 4. Time-dependent changes in HLA-A24-restricted CMV-pp65-specific cytotoxic T-cells (CMV-CTLs) within individual patients between the early and late phases of allo-HCT.

(a) Correlation between the proportions of CMV-CTLs to CD8 T-cells and Shannon's equitability index in the early phase of allo-HCT (P=0.56 by Pearson's correlation test). (b) Proportions of CMV-CTLs in the early phase of allo-HCT according to the CMV-reactivation pattern: no-CMV reactivation (n=10), one-episode (n=9), and repeated reactivation (n=7) groups (P=0.68 by the Kruskal-Wallis (K-W) test, and P=0.19 by the Jonckheere-Terpstra (J-T) test for decreasing tendency). (c) Shannon's equitability index in the early phase of allo-HCT according to the CMV-reactivation pattern (P=0.77 by the K-W test, and P=0.24 by the J-T test for increasing tendency). (d) Changes in proportions of CMV-CTLs between the early and late phases of allo-HCT according to the CMV reactivation pattern: no-CMV reactivation (n=9), one-episode (n=6), and repeated reactivation (n=7) groups. (e) Changes in Shannon's equitability index of CMV-CTLs between the early and late phases of allo-HCT according to the CMV reactivation pattern. (f) The proportions of CMV-CTL clones in the donor, one month after allo-HCT, and >one year after allo-HCT in the no-CMV reactivation group with CMV-seropositive donors (Cases1, 2, & 3). Donor clones of Case 3 had been identified by a direct single-cell method and reported in our previous paper. (g) Changes in the proportions of the top 1 clones of CMV-CTLs within individual patients between the early and late phases of allo-HCT (P=0.48 by the Jonckheere-Terpstra (J-T) test for decreasing tendency). (h) The proportions of CMV-CTL clones that were observed in the early phase but disappeared in the late phase of allo-HCT. Their proportions increased in the following order: no-CMV reactivation < one-episode < repeated CMV reactivation groups (P=0.026 by the J-T test for increasing tendency). (i) The proportions of the newly-appeared CMV-CTL clones in the late phase of allo-HCT. No significant difference was observed according to the CMV-reactivation pattern (P=0.40 by the J-T test for increasing tendency). Individual box and whisker plots were constructed by the 25th percentile (Q1), median, and 75th percentile (Q3) with whiskers of 1.5 times interquartile range (IQR) lengths.

Supplementary Figure 5.

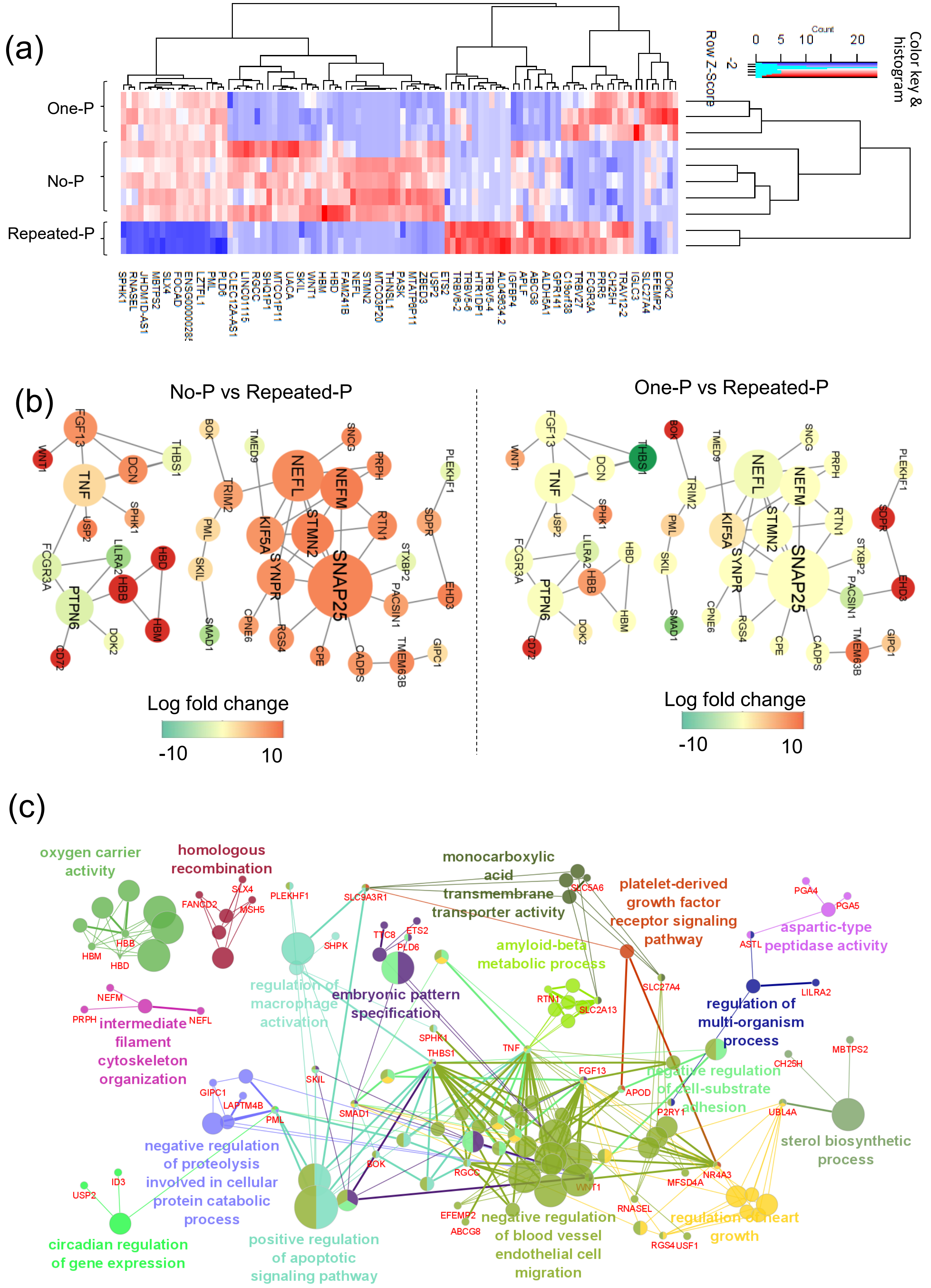

Supplementary Figure 5. Gene expression profile (GEP), protein-protein interaction (PPI) network, and gene ontology (GO) enrichment analyses in HLA-A24-restricted CMV-pp65-specific cytotoxic T-cells (CMV-CTL) according to the CMV reactivation pattern in the early phase of allo-HCT, focusing on the CMV-seropositive donor cohort.

(a) A clustering heatmap of GEP using the top 100 differentially expressed genes (DEGs) according to the CMV-reactivation pattern: no-CMV reactivation (no-P group, n=5), one-episode of CMV reactivation (one-P group, n=3), and repeated episodes of CMV reactivation (repeated-P group, n=2). (b) PPI network constructed by the top 200 DEGs with FDR of <0.05 which had close connections with each other. The shape size suggests the degree of centrality of the PPI network, meaning how many edges are connected to each gene node. The color heat denotes the log-fold changes of the individual gene expression between the no-P vs. repeated-P groups or between the one-P vs. repeated-P groups. Only networks with ≥ 5 connections are shown. (c) GO and the shared genes derived from the top 200 DEGs. Each circle denotes an identified term with a P-value of <0.05 without the Bonferroni correction. The same colors mean GO terms that belong to the same GO term-tree groups. Only the names of the leading GO terms with the highest significance in each GO term-tree group are shown.

Supplementary Figure 6.

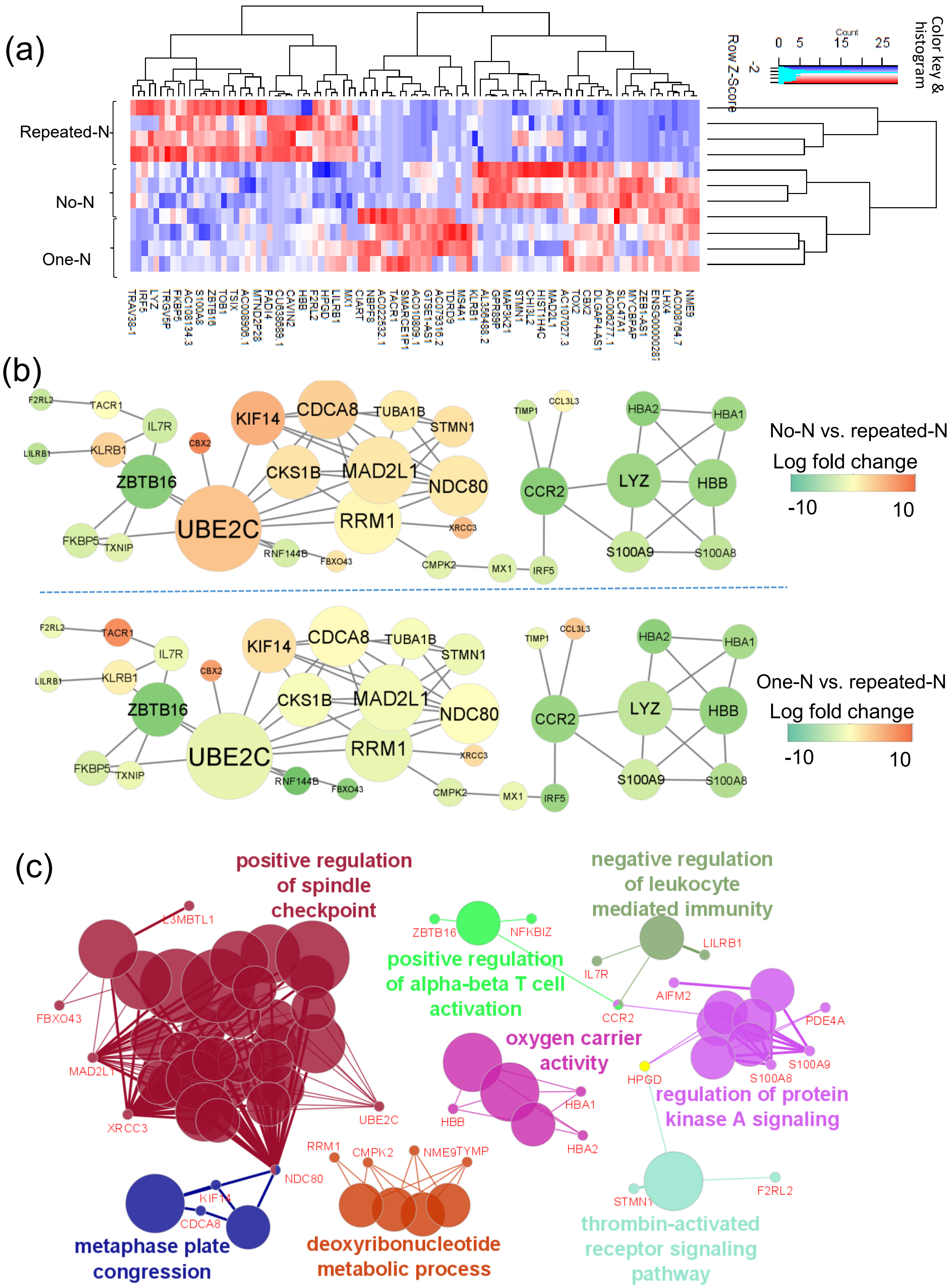

Supplementary Figure 6. Gene expression profile (GEP), protein-protein interaction (PPI) network, and gene ontology (GO) enrichment analyses in HLA-A24-restricted CMV-pp65-specific cytotoxic T-cells (CMV-CTL) according to the CMV reactivation pattern in the early phase of allo-HCT, focusing on the CMV-seronegative donor cohort.

(a) A clustering heatmap of GEP using the top 100 differentially expressed genes (DEGs) according to the CMV-reactivation pattern: no-CMV reactivation (no-N group, n=3), one-episode of CMV reactivation (one-N group, n=4), and repeated episodes of CMV reactivation (repeated-N group, n=4). (b) PPI network constructed by the top 120 DEGs with FDR of <0.15 which had close connections with each other. The shape size suggests the degree of centrality of the PPI network, meaning how many edges are connected to each gene node. The color heat denotes log-fold changes in the individual gene expression between the no-N vs. repeated-N groups or between the one-N vs. repeated-N groups. Only networks with ≥ 5 connections are shown. (c) GO and the shared genes derived from the top 200 DEGs. Each circle denotes an identified term with a P-value of <0.05 without the Bonferroni correction. The same colors mean GO terms that belong to the same GO term-tree groups. Only the names of the leading GO terms with the highest significance in each GO term-tree group are shown.

Supplementary Figure 7.

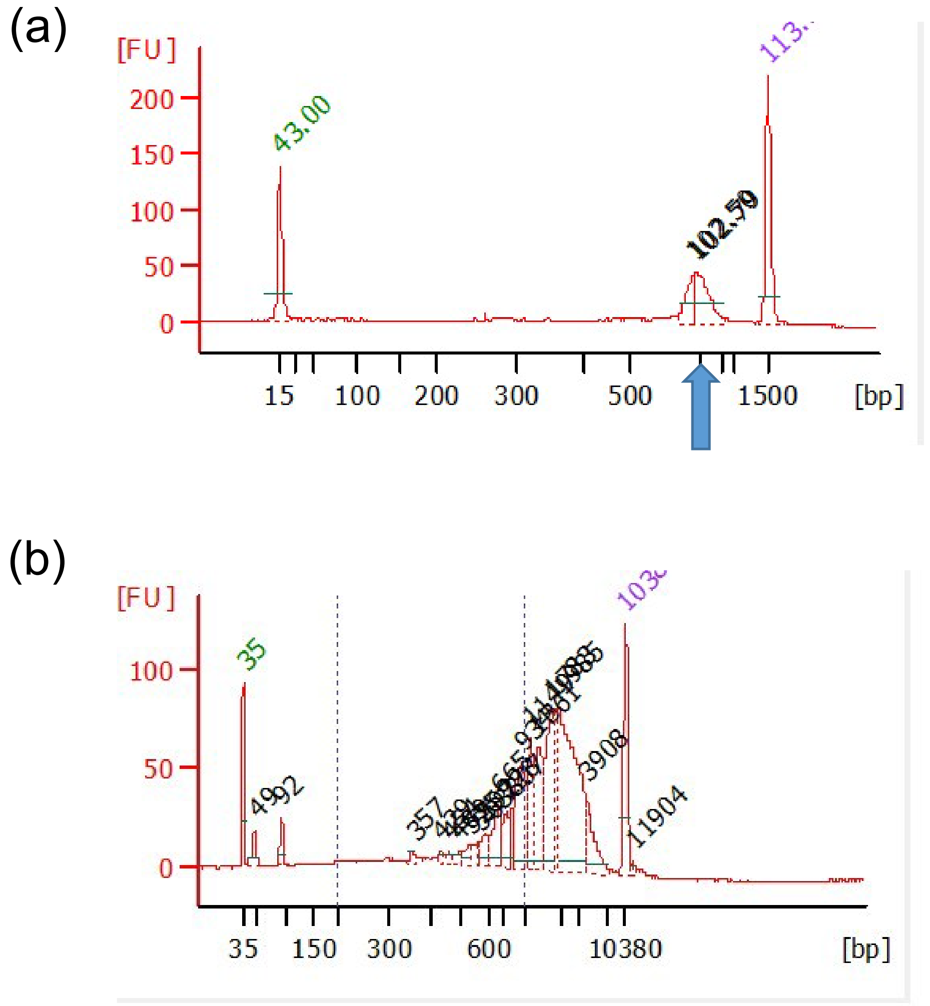

Supplementary Figure 7. Representative figures for quality check of the generated cDNA libraries by electrophoresis using Bioanalyzer. (a) cDNA libraries for T-cell receptor determination. If a peak was detected in the range around 700–800 bp (blue arrows), the synthesized cDNA libraries were transferred to Takara Bio, Inc.(Otus, Japan) for sequence. (b) cDNA libraries for RNA-seq. If a sufficient yield of cDNA was achieved with a distinct peak spanning 400 bp to 10,000 bp, the synthesized cDNA were transferred to Takara Bio, Inc. (Otsu, Japan) for sequence.
