# Supplementary material for: Features of repertoire diversity and gene expression in human cytotoxic T cells following allogeneic hematopoietic cell transplantation
Source: Commun Biol. 2021 Oct 11;4:1177. doi: 10.1038/s42003-021-02709-7 (PMC8505416; doi:10.1038/s42003-021-02709-7)
Supplement: Supplementary file 3 — Description of Additional Supplementary Files [file 42003_2021_2709_MOESM3_ESM.pdf]

Description of Additional Supplementary Files

File name: Supplementary Data

Description: Source data for main Figures.
