# Supplementary material for: Features of repertoire diversity and gene expression in human cytotoxic T cells following allogeneic hematopoietic cell transplantation
Source: Commun Biol. 2021 Oct 11;4:1177. doi: 10.1038/s42003-021-02709-7 (PMC8505416; doi:10.1038/s42003-021-02709-7)
Supplement: Supplementary file 5 — Reporting Summary [file 42003_2021_2709_MOESM5_ESM.pdf]

nature research

Corresponding author(s): Hideki Nakasone

Last updated by author(s): Aug 17, 2021

Reporting Summary

Nature Research wishes to improve the reproducibility of the work that we publish. This form provides structure for consistency and transparency in reporting. For further information on Nature Research policies, see our Editorial Policies and the Editorial Policy Checklist.

Statistics

For all statistical analyses, confirm that the following items are present in the figure legend, table legend, main text, or Methods section.

n/a	Confirmed	
☐	☒	The exact sample size (*n*) for each experimental group/condition, given as a discrete number and unit of measurement
☐	☒	A statement on whether measurements were taken from distinct samples or whether the same sample was measured repeatedly
☐	☒	The statistical test(s) used AND whether they are one- or two-sided<br>*Only common tests should be described solely by name; describe more complex techniques in the Methods section.*
☒	☐	A description of all covariates tested
☐	☒	A description of any assumptions or corrections, such as tests of normality and adjustment for multiple comparisons
☐	☒	A full description of the statistical parameters including central tendency (e.g. means) or other basic estimates (e.g. regression coefficient) AND variation (e.g. standard deviation) or associated estimates of uncertainty (e.g. confidence intervals)
☐	☒	For null hypothesis testing, the test statistic (e.g. *F*, *t*, *r*) with confidence intervals, effect sizes, degrees of freedom and *P* value noted<br>*Give P values as exact values whenever suitable.*
☒	☐	For Bayesian analysis, information on the choice of priors and Markov chain Monte Carlo settings
☒	☐	For hierarchical and complex designs, identification of the appropriate level for tests and full reporting of outcomes
☐	☒	Estimates of effect sizes (e.g. Cohen's *d*, Pearson's *r*), indicating how they were calculated

Our web collection on statistics for biologists contains articles on many of the points above.

Software and code

Policy information about availability of computer code

Data collection	BD FACSDiva v6.1.3. , MiSeq Control Software (MCS) v2.6.2.1, NovaSeq Control Software v1.6.0, Cell Ranger ver3.02
Data analysis	Microsoft Excel 2016, R (ver.4.0, ver4.0.3), EZR (ver1.42, ver1.54), MiSeq Control Software (MCS) v2.6.2.1, Real Time Analysis (RTA) v1.18.54, bcl2fasq2 v2.17, NovaSeq Control Software (v1.6.0, v1.7.0), RTA v3.4.4, bcl2fasq2 v2.20, DRAGEN Bio-IT platform (v3.6.3), Cell Ranger ver3.02 , Loupe Cell Browser (ver.4.0.0) and Loupe VDJ Browser (ver.3.0.0). WebLogo online service (https://weblogo.berkeley.edu/logo.cgi), ERGO system (pEptide tcR matchinG predictiOn) (http://tcr.cs.biu.ac.il/), I-TASSER system (Iterative Threading ASSEmbly Refinement) (https://zhanglab.ccmb.med.umich.edu/I-TASSER/), STRING (Search Tool for the Retrieval of Interacting Genes/Proteins, ver.11.0, http://string-db.org/), Cytoscape (ver.3.8.2), ClueGO (ver.2.5.8) and CluePedia (ver.1.5.8).

For manuscripts utilizing custom algorithms or software that are central to the research but not yet described in published literature, software must be made available to editors and reviewers. We strongly encourage code deposition in a community repository (e.g. GitHub). See the Nature Research guidelines for submitting code & software for further information.

Data

Policy information about availability of data

All manuscripts must include a data availability statement. This statement should provide the following information, where applicable:

- Accession codes, unique identifiers, or web links for publicly available datasets
- A list of figures that have associated raw data
- A description of any restrictions on data availability

All data associated with this study are present in the paper or the Supplementary Materials. Gene expression profile data have been deposited in the GEO database under the accession number (GSE156383).

Field-specific reporting

Please select the one below that is the best fit for your research. If you are not sure, read the appropriate sections before making your selection.

- [x] Life sciences
- [ ] Behavioural & social sciences
- [ ] Ecological, evolutionary & environmental sciences

For a reference copy of the document with all sections, see nature.com/documents/nr-reporting-summary-flat.pdf

Life sciences study design

All studies must disclose on these points even when the disclosure is negative.

Sample size	No calculation of sample size was performed, and the sample size was determined by the availability of samples from actual patients who received allogeneic hematopoietic cell transplantation.
Data exclusions	In the single-cell RNA-seq analyses, the t-SNE clustering plots were shown after deleting apparently dying cells from Case3.
Replication	No replication, since the samples were obtained form actual patients.
Randomization	Randomization was not applicable, since the samples were grouped according to their CMV reactivation patterns and donor CMV seropositivity.
Blinding	The investigators were not blinded to the group allocation as the same investigators both planned and performed the experiment.

Reporting for specific materials, systems and methods

We require information from authors about some types of materials, experimental systems and methods used in many studies. Here, indicate whether each material, system or method listed is relevant to your study. If you are not sure if a list item applies to your research, read the appropriate section before selecting a response.

Materials & experimental systems

n/a	Involved in the study	
[ ]	[x]	Antibodies
[x]	[ ]	Eukaryotic cell lines
[x]	[ ]	Palaeontology and archaeology
[x]	[ ]	Animals and other organisms
[ ]	[x]	Human research participants
[x]	[ ]	Clinical data
[x]	[ ]	Dual use research of concern

Methods

n/a	Involved in the study	
[x]	[ ]	ChIP-seq
[ ]	[x]	Flow cytometry
[x]	[ ]	MRI-based neuroimaging

Antibodies

Antibodies used	T-Select HLA-A*24:02 CMV pp65 Tetramer-QYDPVAALF-PE (TS-0020-1C, Medical & Biological Laboratories)<br>anti-human CD3-FITC(317306, OKT3, BIoLegend) or -APC(317318, OKT3, BIoLegend), CD8a-APC(301014, RPA-T8, BIoLegend) or -FITC (301006, RPA-T8, BIoLegend), CD45RA-PECy7(304128, HI100, BIoLegend), CCR7-APCCy7 (353226, G043H7, BIoLegend) and 7AAD (420404, BIoLegend)
Validation	T-Select HLA-A*24:02 CMV pp65 Tetramer-QYDPVAALF-PE (TS-0020-1C, MBL): Class=Class I, Animal species=Human, Category=virus, Conjugate=PE, Immunogen (Antigen)=CMV pp65, Sequence=QYDPVAALF, MHC Allele=A*24:02, Location (aa) 341-349, Product category & Research area = Immunology &MHC tetramer, data sheet =(https://ruo.mbl.co.jp/bio/dtl/dtlfiles/TS-0020-1C2C-v1-E.pdf)<br>anti-human CD3-FITC(317306, OKT3, BIoLegend): Reactivity=Human, Antibody Type=Monoclonal, Host Species=Mouse, data sheet = (https://www.biolegend.com/en-us/global-elements/pdf-popup/fitc-anti-human-cd3-antibody-3644?filename=FITC%20anti-human%20CD3%20Antibody.pdf&pdfgen=true)<br>CD3-APC (317318, OKT3, Biolegend): Reactivity=Human, Antibody Type=Monoclonal, Host Species=Mouse, data sheet= (https://www.biolegend.com/ja-jp/global-elements/pdf-popup/apc-anti-human-cd3-antibody-6198?filename=APC%20anti-human%20CD3%20Antibody.pdf&pdfgen=true)<br>CD8a-APC(301014, RPA-T8, BIoLegend): Reactivity=Human, Cross-Reactivity=Chimpanzee, Baboon, Cynomolgus, Rhesus, Pigtailed Macaque, Sooty Mangabey, Antibody Type=Monoclonal, Host Species=Mouse, data sheet = (https://www.biolegend.com/en-us/global-elements/pdf-popup/apc-anti-human-cd8a-antibody-831?filename=APC%20anti-human%20CD8a%20Antibody.pdf&pdfgen=true)<br>CD8a-FITC(301006, RPA-T8, BIoLegend): Reactivity=Human, Cross-Reactivity=Chimpanzee, Baboon, Cynomolgus, Rhesus, Pigtailed Macaque, Sooty Mangabey, Antibody Type=Monoclonal, Host Species Mouse, data sheet =(https://www.biolegend.com/ja-jp/global-elements/pdf-popup/fitc-anti-human-cd8a-antibody-834?filename=FITC%20anti-human%20CD8a%20Antibody.pdf&pdfgen=true)<br>CD45RA-PECy7(304128, HI100, BIoLegend): Reactivity=Human, Cross-Reactivity=Chimpanzee, Antibody Type=Monoclonal, Host Species=Mouse, data sheet = (https://www.biolegend.com/en-us/global-elements/pdf-popup/apc-cyanine7-anti-human-cd45ra-

antibody-7056?filename=APCCyanine7%20anti-human%20CD45RA%20Antibody.pdf&pdfgen=true)
CCR7-APCCy7 (353226, G043H7, BIoLegend): Reactivity=Human, African Green, Baboon, Cynomolgus, Rhesus, Antibody Type=Monoclonal, Host Species=Mouse, Immunogen=CCR7-transfected cells, data sheet = (https://www.biolegend.com/en-us/global-elements/pdf-popup/pecyanine7-anti-human-cd197-ccr7-antibody-7694?filename=PECyanine7%20anti-human%20CD197%20CCR7%20Antibody.pdf&pdfgen=true)
7AAD (420404, BIoLegend): Application Notes=7-AAD Viability Staining Solution can be used as a viability probe for methods of nonviable cell exclusion. data sheet = (https://www.biolegend.com/en-us/global-elements/pdf-popup/7-aad-viability-staining-solution-1649?filename=7-AAD%20Viability%20Staining%20Solution.pdf&pdfgen=true)

Human research participants

Policy information about studies involving human research participants

Population characteristics	Patient characteristics were described in Table 1. Briefly, this study included CMV-seropositive recipients who received allo-HCT in our institution , and who survived for ≥6 months without disease relapse. All of their corresponding donors had to have HLA-A24:02 or -A24:20.
Recruitment	Among transplant recipients who gave their written informed consent to participate the studies of immune reconstitution after allo-HCT, we selected those who met the inclusion criteria: CMV-seropositive recipients, ≥6 months survivors without relapse, and heir corresponding donors with HLA-A24:02 or -A24:20. Selection bias might be present based on their sample availability. However, TCR diversity and structure would not be considered to be affected by the sample availability.
Ethics oversight	This study was approved by the institutional review board of Jichi Medical University and all subjects gave their written informed consent for the cryopreservation and analysis of blood samples in accordance with the Helsinki declaration.

Note that full information on the approval of the study protocol must also be provided in the manuscript.

Flow Cytometry

Plots

Confirm that:

- [x] The axis labels state the marker and fluorochrome used (e.g. CD4-FITC).
- [x] The axis scales are clearly visible. Include numbers along axes only for bottom left plot of group (a 'group' is an analysis of identical markers).
- [x] All plots are contour plots with outliers or pseudocolor plots.
- [x] A numerical value for number of cells or percentage (with statistics) is provided.

Methodology

Sample preparation	Cells were spun down, and pellets were re-suspended in PBS or RPMI containing 2-10% of FBS. Discard supernatant, re-suspend the cell pellet, and incubate a at room temperature (RT) in the dark for 30 min after adding 10ul of HLA-A*24-CMV-pp65 (QYDPVAALF)-tetramer(MBL). Then, add anti-human CD3-FITC (or-APC), CD8-APC (or-FITC), CD45RA-PECy7, CCR7-APCCy7 and 7AAD (BioLegend), and incubate in the dark for 25-30 min. Stained PBMCs were subjected to flow cytometry analyses
Instrument	BDFACSAriaII (BD Biosciences)
Software	BD FACSDiva v6.1.3. software
Cell population abundance	No post-sort confirmation was performed because the targeted cell counts were too small (30-1000).
Gating strategy	Doublet cells were excluded by plotting forward scatter (FSC) height vs. FSC area as well as side scatter (SSC) height vs. SSC area. CMV-CTLs were defined as CD3+CD8+CMV-teramer+ T-cells. No other gating strategy was used.

- [x] Tick this box to confirm that a figure exemplifying the gating strategy is provided in the Supplementary Information.
